# Is there a relationship between serum vaspin levels and insulin resistance in chronic renal failure?

**DOI:** 10.12669/pjms.35.1.96

**Published:** 2019

**Authors:** Can Demir, Akif Dogantekin, Ali Gurel, Suleyman Aydin, Huseyin Celiker

**Affiliations:** 1 Can Demir Hayat Hospital, Internal Medicine Department, Gaziantep, Turkey; 2 Akif Dogantekin Emek Hospital, Internal Medicine Department, Gaziantep, Turkey; 3 Ali Gurel Adiyaman University Medical Faculty, Nephrology Department, Adiyaman, Turkey; 4 Suleyman Aydin Department of Biochemistry, Firat University Medical Faculty, Elazig, Turkey; 5 Huseyin Celiker Deparment of Nephrology, Firat University Medical Faculty, Elazig, Turkey

**Keywords:** Chronic kidney disease, Vaspin, Insulin resistance

## Abstract

**Objective:**

Chronic kidney disease (CKD) patients have insulin secretion disorders and resistance to insulin effects, that is responsible for the development of cardiovascular events. Vaspin is an adipocytokine that regulates glucose and lipid metabolism. We aimed to determine the serum vaspin levels and its relationship with insulin resistance in CKD patients.

**Methods:**

In the study groups, serum vaspin levels, anthropometric parameters and routine blood tests were measured. The serum vaspin levels were examined by the enzyme-linked immunosorbent assay (ELISA) and insulin resistance was determined by the homeostasis model assessment of insulin resistance (HOMA-IR) formula.

**Results:**

The serum vaspin, HOMA-IR index and insulin levels were observed significantly high in the CKD group in comparison with the control group. No correlation was found between the serum vaspin level and the anthropometric and metabolic values. The serum vaspin level was positively correlated with the fasting plasma glucose and age but without statistical significance.

**Conclusion:**

Insulin resistance and hyperinsulinemia contribute to the development of cardiovascular complications in CKD. We consider that the increase in the serum vaspin level is a consequence of the reduced renal excretion in the CKD and increases in response to insulin resistance.

## INTRODUCTION

Chronic renal disease is defined as the objective renal damage and/or reduction in the glomerular filtration rate (GFR) of less than 60 mL/min/1.73 m² for at least three months regardless of the underlying etiology of the renal disease.^1^ In the early stages of CKD, only the functional reserve of the kidney is reduced. Increasing uremia in end-stage renal disease (ESRD) reveals signs and symptoms in almost every organ system defined as the “uremic syndrome”.^2^ The most common causes of CKD are diabetes mellitus (DM), hypertension, chronic glomerulonephritis, all over the world. Renal replacement therapy should be performed for patients with ESRD.^2^-^4^

Insulin is a polypeptide hormone produced by the beta cells of the pancreas Langerhans islets.^5^ Peripheral insulin resistance can be defined as a condition in which the normal response to exogenous or endogenous insulin is impaired, and consequently the amount of insulin required for the normal functions of the cells, tissues, or organism increases.^6^-^8^

Homeostasis model assessment is a method of evaluating β-cell function and insulin resistance according to basal glucose, insulin, or C-peptide concentrations.^9^ Kidneys play an important role in insulin metabolism and clearance, and they are target organs in insulin metabolism disorders at the same time. All of the exogenous insulin and 30%–80% of the endogenous insulin are metabolized in the kidney. The significant clinical changes in glucose metabolism in uremic patients are insulin resistance, impaired insulin secretion, increased glyconeogenesis, decreased insulin and glucagon disintegration.^10^

Adipose tissue is the largest source of energy in the body. This energy is stored in the form of triglycerides, which can rapidly go through circulation as fatty acids in case of hunger or when needed. Hormones such as insulin, adrenaline, noradrenaline, and cortisone act on adipocytes and regulate their function. Adipose tissue also functions as an endocrine organ.^11^ Adipocytokines can be divided into two groups: “insulin resistance inducing factors,” such as resistin, TNF-α, and interleukin 6 (IL-6), and “insulin sensitivity inducing factors,” such as leptin, adiponectin, apelin, and the recently identified visfatin.^12^ These cytokines are considered to play a role in the etiopathogenesis of insulin resistance and cardiovascular events.^13^,^14^ Vaspin (visceral adipose tissue-derived serpin) is a novel adipocytokine that regulates glucose and lipid metabolism.^15^ It is a member of the serine protease family and is secreted in Otsuka Long–Evans Tokushima Fatty (OLETF) rats in case of obesity and peak insulin plasma concentrations from the visceral adipose tissue.^16^ Vaspin is considered a novel biomarker that is potentially effective against obesity and impaired insulin tolerance, and it is released as an antiprotease factor in visceral adipose tissue; clinically worsening diabetes and weight loss decrease vaspin expression. In a study involving diabetic patients, serum vaspin levels were correlated with insulin resistance and HbA1c and were found to be lower in women with microvascular complications than in those without complications.^17^

## METHODS

Twenty-six predialytic patients (stage 3-5 CKD) and 29 healthy volunteers were included in the study. All of the participants were informed before the study, and their written consents were obtained. Patients with systemic diseases such as infection, malignant disease, DM, obesity (BMI > 30 kg/ m^2^), and treatment with anti-inflammatory agents and antioxidants were excluded from the study. The BMI values of the patients were recorded as kg/m^2^. Venous blood samples were obtained from all patients following a 12 h fasting for biochemical and immunological analyses. A total of 5 cc of blood taken for the serum vaspin level was stored in the tube containing aprotinin for 10 min and then centrifuged at 4,000 rpm for 5 min. The obtained serum was stored at −20 °C in the deep freezer until needed. The collected samples were solved in the laboratory environment and analyzed with Human Vaspin ELISA Kit (ALPCO Immunoassays, Catalog Number: 44-VASHUE01) according to the manufacturer’s instructions in an ELISA reader (BIOTEC, EL: 800) and recorded as ng/mL.

Insulin resistance was calculated as HOMA-IR = fasting insulin (μu/ml) × fasting glucose (mmol/L) / 22.5. The GFR was calculated with the MDRD formula as GFR = 170 × (serum Cr) −0.999 × (age) −0.176 × (0.762 for females) × (1.180 for black patients) × (serum BUN) −0.170 × [(Alb) + 0.318].

**Fig.1a:**
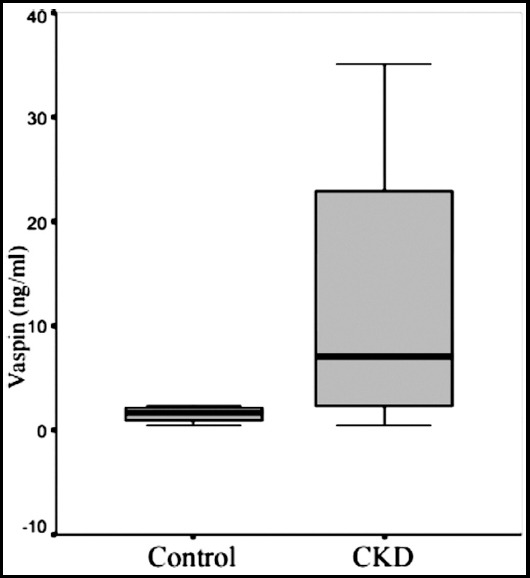
Mean serum vaspin levels of groups.

**Fig.1b:**
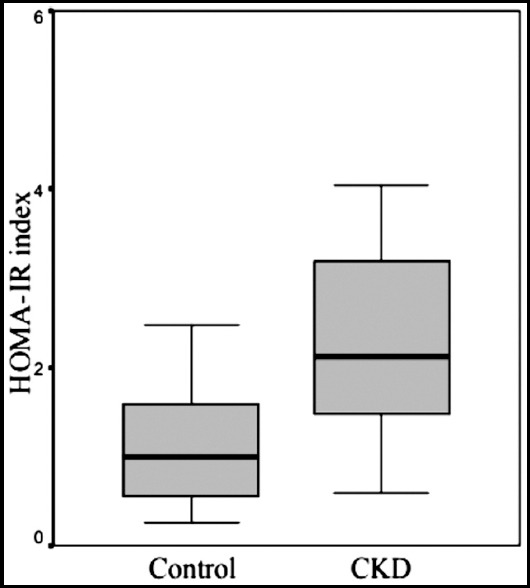
Mean HOMA-IR indexes of groups.

**Fig.1c:**
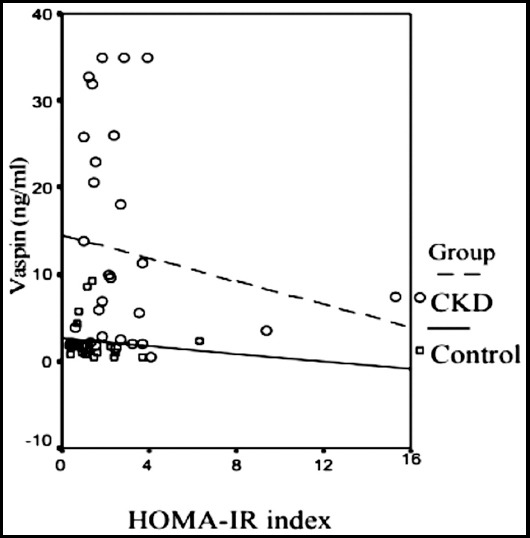
Relationship between serum vaspin level and HOMA-IR of groups (Control group: p: 0.576, r: -0.130, CKD group: p: 0.412 r: -0.155)

Statistical analysis was performed using the Statistical Package for Social Sciences program version 12.0. The parametric data were expressed as mean ± standard deviation, and the non-parametric data were expressed as (%). Student’s t-test was used to compare the parametric data, and Pearson’s correlation test was employed to determine the correlation between the parameters. Normalization of distribution was assessed by the Kolmogorov–Smirnov test, and logarithmic transformations were applied if necessary. The results were evaluated at a confidence interval of 95% and a significance level of p < 0.05.

## RESULTS

The cases were divided into two groups as the control and the CKD group. When the groups were compared in terms of demographic characteristics, no statistically significant difference (p > 0.05) was found (Table-I).

**Table-I:** Demographic characteristics of groups.

	Control (n=22)	CKD (n=33)
Age (year)	48.4±15	53.8±15.7
Sex (F/M)	14/8 (%)	14/19 (%)
WAIST (cm)	87.1±13.6	90.9±12.2
Height (cm)	165.4±7.4	163.9±9.9
Weight (kg)	70.5±10.4	69.5±11.8
BMI (kg/m^2^)	25.8±3.8	25.9±3.9

BMI: Body Mass Index, F: Female, M: Male.

The serum glucose and HbA1c levels were not significantly different between the groups. However, HOMA-IR index, insulin, C-peptide and vaspin levels were significantly higher in the CKD group than in the control group (p < 0.05). A significant correlation was found between serum vaspin levels and HbA1c in the CKD group (p < 0.05). A correlation was also found between serum vaspin levels and age and serum glucose levels but without statistical significance. In the CKD group, serum glucose levels were negatively correlated with high-density lipoprotein (HDL) and positively correlated with body weight (p < 0.05). A statistically significant positive correlation was observed between HOMA-IR and triglyceride levels in the CKD group (p < 0.05). No significant difference was found in serum vaspin levels between patients with and those without insulin resistance in the CKD group. In the control group, a statistically significant correlation was found between triglyceride levels and waist circumference and body weight (p<0.05). A significant correlation was found between waist circumference and insulin levels, triglyceride levels, and low-density lipoprotein (LDL) (p<0.05) as well as between HOMA-IR and the LDL in control group (p < 0.05). Table-IIa shows the mean values of serum glucose, insulin, HbA1c, and HOMA-IR index of the groups.

**Table-IIa:** Mean values of serum glucose, insulin, HbA1c levels, HOMA-IR index and vaspin levels of the groups.

	Control (n=22)	CKD (n=33)
FPG (mg/dl)	84.4±11.7	87.6±10.6
Insulin (mU/L)	6.9±5.6	13.3±2.5^b^
e-peptid	1.9±1.0	4.2±2.2^a^
HOMA-IR	1.5±1.4	2.8±2.7^a^
HbA_1_c (%Hb)	5.28±0.5	5.37±0.4
Vaspin (ng/ml)	2.4±2.5	12.6±12.2^a^

BMI: Body Mass Index, F: Female, M: Male.

Triglyceride levels were higher and total cholesterol, LDL, and HDL levels were lower in the CKD group than in the control group, but only low HDL levels were significant (p < 0.05) (Table-IIb).

**Table-IIb:** Mean lipid levels of groups.

	Control (n=22)	CKD (n=33)
Total cholesterol (mg/dl)	203.1±47	190.9±39.1
LDL- cholesterol (mg/dl)	121.9±35.7	121.7±30.8
HDL- cholesterol (mg/dl)	54.7±11.8	46.3±13.4^a^
Triglycerides (mg/dl)	140.4±70.0	159.2±69.1

LDL: Low-density lipoprotein, HDL: High-Density Lipoprotein, In comparison with control group:

a p<0.05.

No statistically significant difference was observed in the total protein levels between the groups, but albumin levels in the CKD group were statistically significantly lower than those in the control group (p < 0.05). Urea, creatinine, and parathyroid hormone levels were significantly higher and hemoglobin and hematocrit levels were significantly lower in the CKD group than in the control group (p < 0.001) (Table-IIc).

**Table-IIc:** Mean laboratory values of groups.

	Control (n=22)	CKD (n=29)
Total protein (g/dl)	7.2±0.6	7.3±0.7
Albumin (g/dl)	4.3±0.4	4.0±0.6^b^
Urea (mg/dl)	107±45.8	28±9.2^b^
Creatinine (mg/dl)	0.9±0.1	3.3±1.4^b^
Parathyroid Hormone (mg/dl)	71±25.7	289.5±206.6^a^
Hemoglobin (g/dl)	13.2±1.8	11.9±1.8^b^
Hematocrit (%)	40.7±4.8	36.7±6.5^b^

In comparison with control group:

a p<0.05,

b p<0.001.

## DISCUSSION

Vaspine is an adipocytokine that plays a regulatory role in glucose and lipid metabolism.^15^ Serum vaspin expression decreases with uncontrolled DM and weight loss, whereas serum concentrations increase with obesity.^17^,^18^ Vaspin levels can be normalized by insulin and pioglitazone therapy.^16^ As serum vaspin concentrations correlate with obesity and tests for body fat distribution, vaspin may be considered a new cytokine that may contribute to atherosclerosis and obesity.^18^

Gulcelik et al.^3^ found a positive correlation between serum vaspin levels and HbA1c in 37 type 2 DM and 37 control Turkish women with similar age and BMI as well as a lower serum vaspin levels in patients with an HbA1c level of less than 7% than in those with greater than 7% and in diabetic patients with microvascular complications than in those without. Patients who received metformin treatment had lower vaspin levels than those who did not. In the same study, a positive correlation was observed between serum vaspin levels and HOMA-IR, insulin level, and HbA1c in diabetic patients.

Adipocytokine levels are significantly increased in CKD possibly because of decreased renal excretion^4^. Ramos et al.^19^ and Inoue et al.^20^ found that serum vaspin levels were lower in patients under hemodialysis than in the control group. Szczepanska et al.^21^ also found that serum vaspin levels were lower in children with CKD than in the control group.

Seeger et al.^22^ compared serum vaspin levels in 60 hemodialysis patients and in the control group with a GFR greater than 50 ml/ min and found similar levels in both groups and decreased serum vaspin levels in patients using insulin. In our study, the patients in the CKD group were those with moderate-to-advanced renal failure who did not receive dialysis, whereas in the study of Seeger et al., the serum vaspin levels were found to be as high as 2.4 ± 2.5 ng/ mL in the healthy control group and 12.6 ± 12.2 ng/ mL in the non-diabetic patients with CKD. The difference in the vaspin levels between our two groups was statistically significant, and we attributed this difference to the reduction in renal excretion, possibly as in other adipokines. In our study, a statistically insignificant correlation was found between vaspin and age, and no correlation was observed between serum vaspin level and HOMA-IR and insulin levels in the non-diabetic patients with CKD.

Chang et al.^23^ divided 150 patients into two groups of high and low HOMA-IR levels and investigated the relationship between HOMA-IR level and serum vaspin level. No difference was found between the low and high HOMA-IR groups in terms of serum vaspin levels. In the high HOMA-IR group, serum vaspin concentrations were positively correlated with age and visceral adipose tissue mass. Conversely, serum vaspin concentrations in the low HOMA-IR group and control group were not correlated with any anthropometric and metabolic variables. In our study, the HOMA-IR value of the control group was low (1.5 ± 1.4), and a positive correlation was found between HOMA-IR and LDL level (p < 0.05, r: 0.545). In the CKD group, we found a high HOMA-IR value (2.8 ± 2.7). Despite the significantly higher serum vaspin level, we did not observe a correlation between vaspin and HOMA-IR. A positive correlation between fasting plasma glucose and vaspin was observed, but without statistical significance possibly because of the small number of sample size. In our study, when we divided the groups into low and high HOMA-IR, no statistically difference was observed in the serum vaspin levels in both groups.

Hida et al.^16^ found decreased serum vaspin levels with worsening diabetes in OLEFT rats. Tan et al.^24^ examined the vaspin level and mRNA expression in omental adipose tissue and in women with PCOS and found high serum and adipose tissue vaspin levels. In addition, they found a positive correlation between serum and adipose tissue vaspin levels and waist-to-hip ratio and BMI. In patients with PCOS, the reduction in insulin resistance due to six months of metformin therapy was accompanied by decreased serum vaspin and glucose levels. The authors hypothesized that the increase in vaspin levels in serum and adipose tissue could be due to the compensatory response to insulin resistance.^3^,^24^ In uremic patients without diabetes, insulin resistance was found when the GFR fell below 50 ml/min.^10^,^25^ In our study, the patients in the CKD group were those with stage 3-5 renal insufficiency due to non-diabetic causes. Similar to the literature, the HOMA-IR score was significantly higher in the CKD group than in the control group (p < 0.001). A positive correlation was observed between HOMA-IR score and triglyceride level in the CKD group (p < 0.05, r: 0.371). No correlation was found between HOMA-IR and BMI in both groups.

## CONCLUSION

In this study, a direct correlation between serum vaspin level and HOMA-IR and insulin levels, which are indicators of insulin resistance, could not be determined in the CKD group. Insulin resistance and serum vaspin levels in the CKD group were significantly higher than those in the control group. A positive correlation was found between HOMA-IR and insulin and triglyceride levels in the CKD group, and a statistically insignificant correlation was observed between serum vaspin level and age and fasting plasma glucose. Whether elevated serum vaspin levels increase as a preventive measure against increased cardiovascular risk in patients with CKD is not known. Increased vaspin levels are considered to be associated with increased insulin resistance, uremic toxins, and multifactorial factors in CKD. Other reasons, such as decreased renal excretion and chronic inflammatory condition, may be effective in increased serum vaspin levels in patients with CKD. The low number of patients studied could have affected the unclear correlation between insulin resistance and serum vaspin levels.

### Authors Contribution

**CD:** Designed the study and collected specimens.

**AD:** Did statistical analysis.

**AG:** Collected specimens and did writing and editing of manuscript.

**SA:** Did laboratory analysis of specimens.

**HÇ:** Did review of manuscript.
